# Infection of Cultured Human and Monkey Cell Lines with Extract of Penaeid Shrimp Infected with Taura Syndrome Virus

**DOI:** 10.3201/eid0902.020181

**Published:** 2003-02

**Authors:** Josefina Audelo-del-Valle, Oliva Clement-Mellado, Anastasia Magaña-Hernández, Ana Flisser, Fernando Montiel-Aguirre, Baltasar Briseño-García

**Affiliations:** * Universidad de Occidente, Los Mochis, Sinaloa, México; † Instituto de Diagnóstico y Referencia Epidemiológicos, México, D.F., México; ‡ Universidad Nacional Autónoma de México, México, D.F., México

**Keywords:** mammalian cultured cells, picornavirus superfamily, shrimp viral disease, Taura syndrome virus, dispatch

## Abstract

Taura syndrome virus (TSV) affects shrimp cultured for human consumption. Although TSV is related to the Cricket Paralysis virus, it belongs to the “picornavirus superfamily,” the most common cause of viral illnesses. Here we demonstrate that TSV also infects human cell lines, which may suggest that *Penaeus* is a potential reservoir of this virus.

The Taura syndrome virus (TSV) causes a disease affecting penaeid shrimp, the most important commercial family of crustaceans (*1*). The causal agent is a single-stranded (+) RNA virus, recently reported to be genomically related to the Cricket Paralysis virus of the *Cripavirus* genus, family *Dicistroviridae* of the “picornavirus superfamily” (*2*–*5*). This superfamily includes several human pathogens, for example, the common cold viruses and several enteroviruses (e.g., polioviruses). Traditionally, research on the replication of shrimp viruses has been based on the use of cultured fish cellular lines (*6*). However, because TSV could potentially represent a public health threat, we explored whether this viral agent might be capable of infecting cultured mammalian cells.

## The Study

Since Sabin strain LSc 2ab (Sabin 1), the poliovirus used for human vaccination, is usually replicated in monolayer culture cells of human rhabdomyosarcoma (RD), human larynx carcinoma (Hep-2C) (), or Buffalo green monkey kidney (BGM) (*7*), we injected these cell lines with a 0.22-µm membrane-filtered whole extract of the hepatopancreas of shrimp (*Penaeus stylirostris*) affected with TSV. The animals were collected from farms located in northwestern Mexico. Control cell lines were injected with filtered hepatopancreas extracts from healthy shrimp. Cultures were incubated at 37°C and periodically observed under a microscope until any sign of cytopathic effect was detected (usually from 19–23 hours). Cells were then harvested and lysed. Fresh cell lines were inoculated with the lysate, incubated, and processed in a similar way. A third inoculation was again performed with the second lysate (*8*).

**Figure 1:**
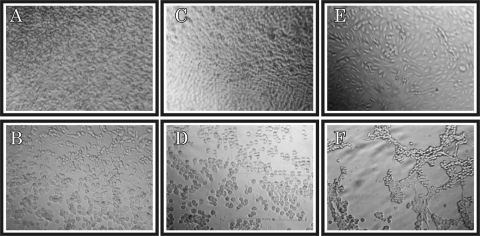
Image of mammalian cell lines injected with extracts from healthy shrimp: A, human rhabdomyosarcoma (RD), Cep-2C, BGM RD cells; C, human larynx carcinoma (Hep-2C) cells; E, BGM cells. Cytophatic effect in cultured cells inoculated with extracts from shrimp affected with Taura syndrome: B, RD cells; D, Hep-2C cells; F, BGM cells.

The cytopathic effect in RD cells began with a partial destruction of the cellular layer. Next, small cellular groups and some isolated round cells were observed. The cells showed an apparent increase in size, diffuse cell rounding, and a refringent aspect (Figure 1B). In Hep-2C cells, the cellular monolayer was partially destroyed. Most cells were individualized and clearly rounded; they also presented a refringent aspect. Hep-2C was the most affected of the three lines used (Figure 1D). The cytopathic effect in the BGM cell line began as a partial destruction of the cellular layer, which evolved to a syncytial-like formation of rounded, refringent cells. Some cells remained isolated but with altered morphology (Figure 1F). RD, Hep-2C, and BGM cells injected with an extract similarly processed but from healthy shrimp, showed no cytopathic effects, even after 7 days of culture (Figures 1A, 1C, and 1 E). As a positive control, RD cells were injected with Sabin viral extract and showed the characteristic cytopathic effect produced by an enterovirus infection.

**Figure 2:**
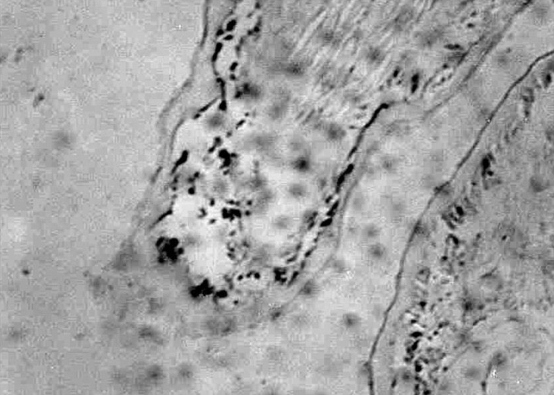
Microscopic image of the subcuticular tissue of the pleopod from a shrimp infected with the supernatant of the third passage of a human larynx carcinoma (Hep-2C) cell culture inoculated with an extract of shrimp infected with Taura syndrome virus. The presence of the virus is clearly visible by in situ hybridization as black spots after the samples were stained with Bismarck brown.

To confirm the presence of TSV in the cell culture media, a bioassay was performed by using media from the third passage. For this assay, healthy *P.* *stylirostris* shrimp were injected with the infected medium in 10% volume of their corporal mass in the third abdominal segment. Twenty-four hours later, these animals were clearly infected, showing fragile antennas and soft cuticle as well as chromatophore expansion along the whole surface of the body, particularly at the tail fan (telson and uropods). These signs were clinically indistinguishable from those occurring in naturally infected animals and are considered as pathognomonic of the acute phase of infection by TSV (*9*). Presence of the viral genome in different subcuticular tissues (gills and pleopods) of these animals was confirmed by in situ hybridization by using TSV ShrimProbe (DiagXotics, Inc., Wilton, CT). RNA-DNA hybrids were clearly visible as black spots after the samples were stained with Bismarck brown (Figure 2). Shrimp injected with culture media from control cell lines showed no signs of infection after 7 days of observation.

## Conclusions

If one takes into consideration the capacity of viruses to modify receptor recognition and host cell tropism and the fact that cell receptors for many of the picornavirus superfamily members seem to be ubiquitous membrane molecules (e.g., decay-accelerating factor, different type of integrins, low-density lipoprotein receptor, sialic acid [*10**–**12*]), the potential wide range of host cells for TSV should not come as a surprise. To our knowledge, these cultured human and monkey cell lines are the first reported to be infected with a viral agent isolated from shrimp. Because many members of the picornavirus superfamily are the most common causes of viral illnesses worldwide (including nonspecific febrile illnesses, myocarditis, aseptic meningitis, and sepsis-like disease), such illnesses lead to frequent unnecessary prescription of antibiotics (*13*). *Penaeus* could be considered as a reservoir of a virus that could become a potential pathogen to humans and other mammals (*11*,*14*).
